# Supplementary material for: Widespread global disparities between modelled and observed mid-depth ocean currents
Source: Nat Commun. 2023 Apr 12;14:2089. doi: 10.1038/s41467-023-37841-x (PMC10097707; doi:10.1038/s41467-023-37841-x)
Supplement: Supplementary file 1 — Supplementary Information [file 41467_2023_37841_MOESM1_ESM.pdf]

Supplementary information for:

Widespread global disparities between modelled and observed mid-depth ocean currents

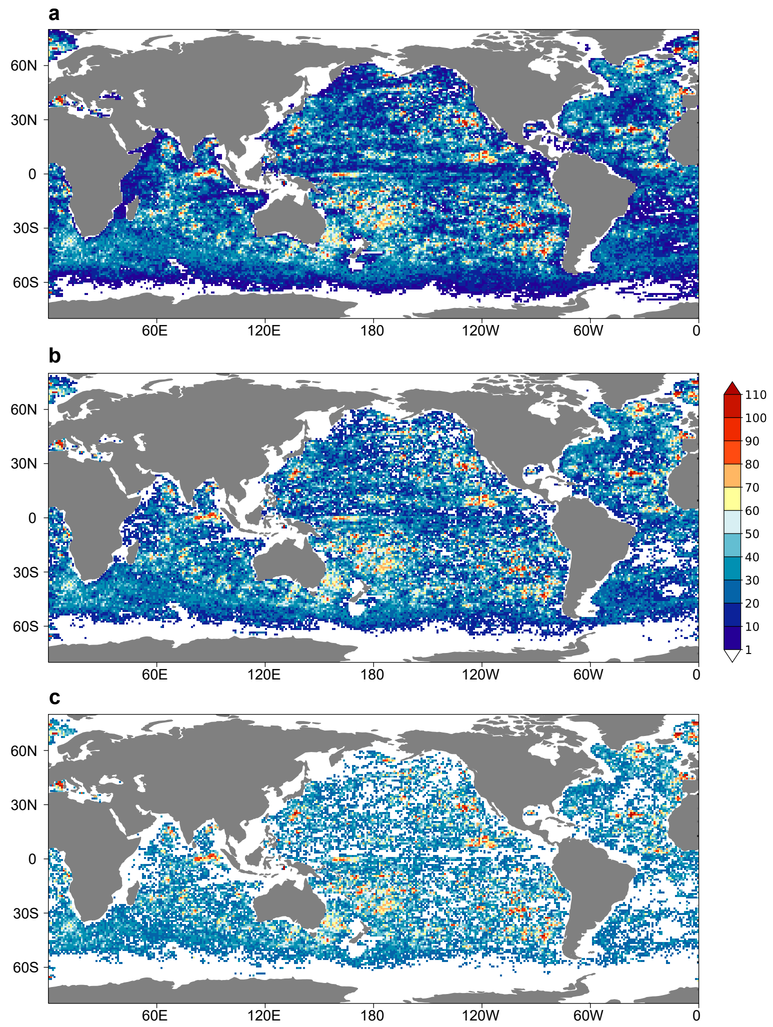

Supplementary Fig. 1 | Spatial distribution of Argo observations. Spatial coverage of ANDRO dataset where there are more than (a) 1 observation, (b) 10 observations, and (c) 20 observations within the respective 1°×1°geogrid.

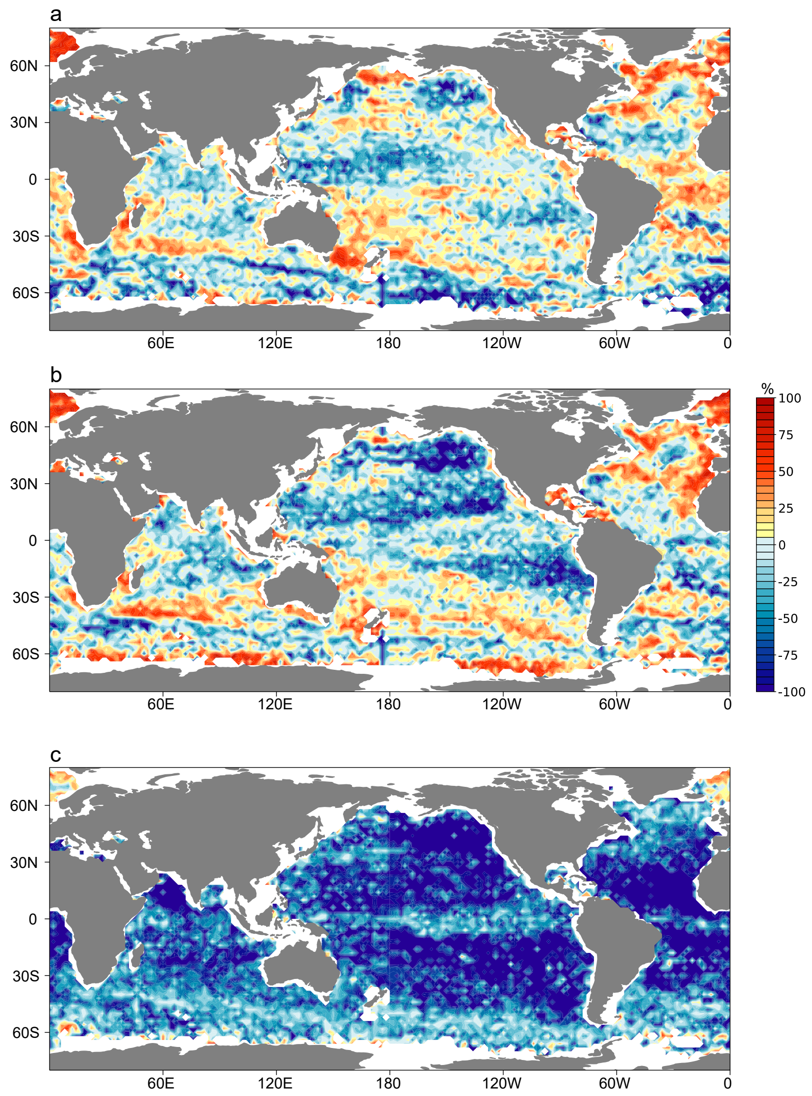

Supplementary Fig. 2 | Global spatial distribution of PDOV (Percentage of velocity difference). (a) ECCO2, (b) OFES, (c) CMEMS. Warm (Cool) color indicates that observed velocity of float displacements exceeds (falls behind) simulated velocity.

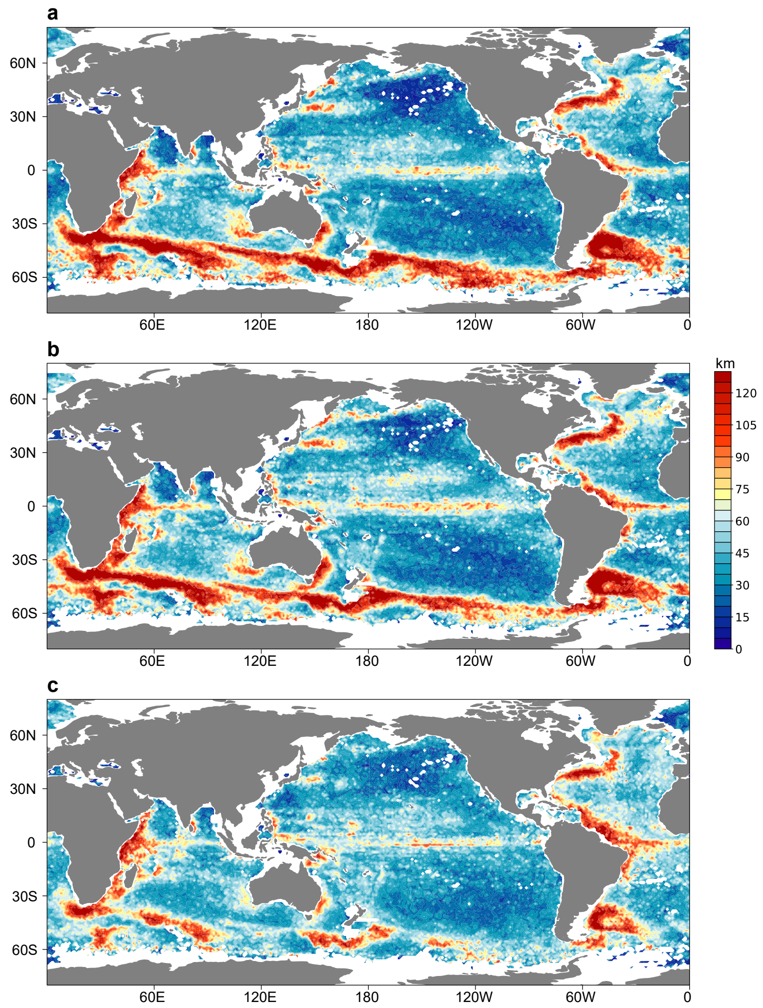

Supplementary Fig. 3 | Global spatial distribution of SD (Separation distance). (a) ECCO2, (b) OFES, (c) CMEMS. The red and blue colors represent the highest and lowest SD values, respectively.

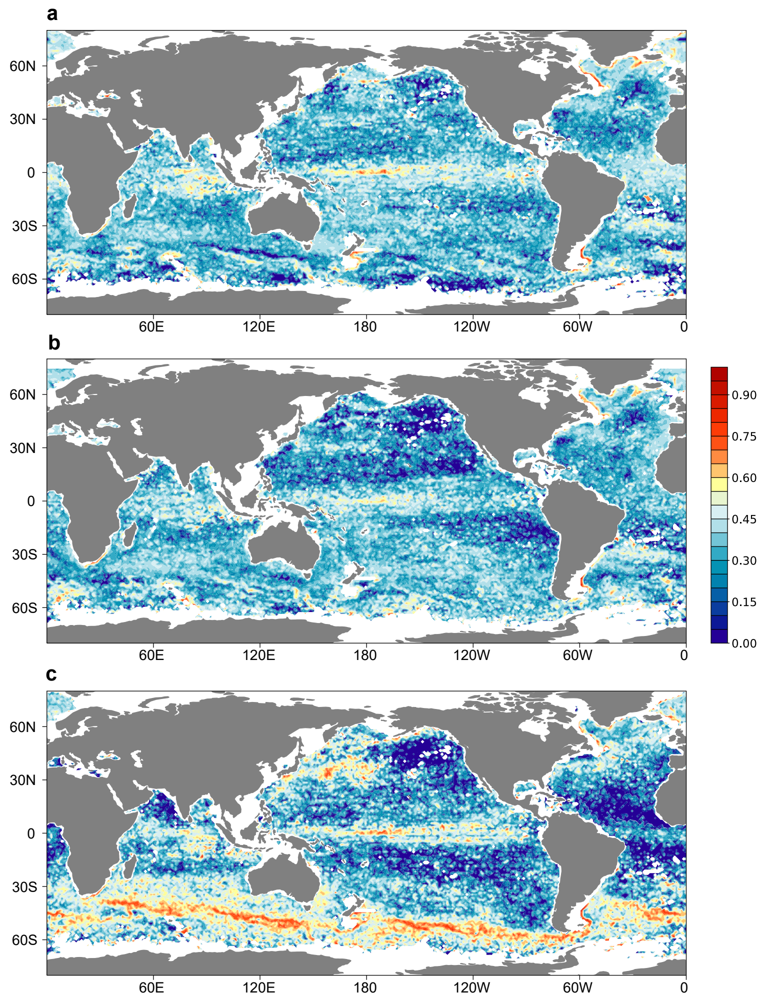

Supplementary Fig. 4 | Global spatial distribution of SS (Skill score). (a) ECCO2, (b) OFES, (c) CMEMS. SS is obtained using n = 1.8 in the indicator calculation. Larger SS values correspond to those localities with a better understanding of current circulation. The red and blue colors represent the highest and lowest SS values, respectively.

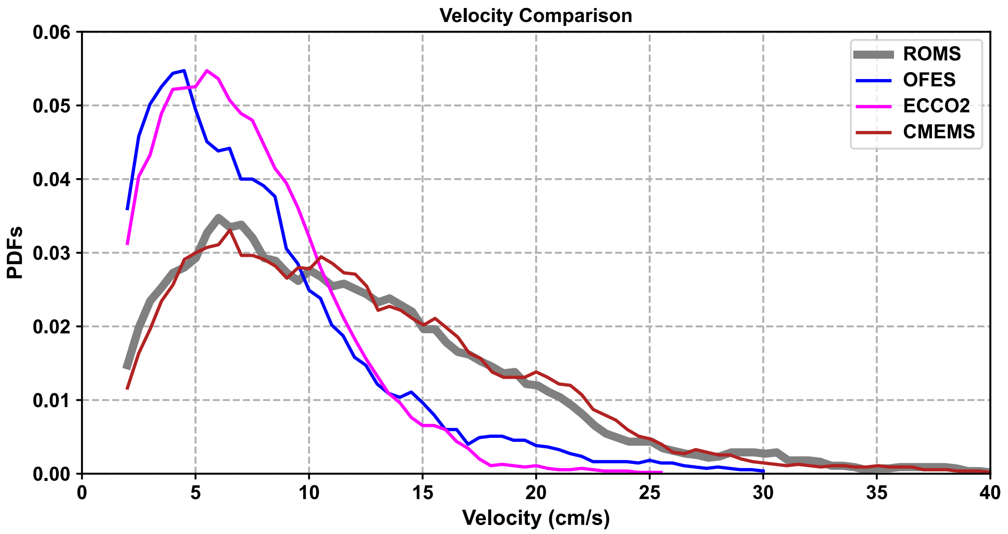

Supplementary Fig. 5 | The probability density function (PDF) of absolute velocity from simulated floats without diffusivity. All floats are deployed only in the Gulf Stream. The grey line represents the simulated floats advected by ROMS velocity fields. Other colored lines represent the simulated floats advected by OFES, ECCO2, CMEMS GLORYS12 velocity fields, respectively.

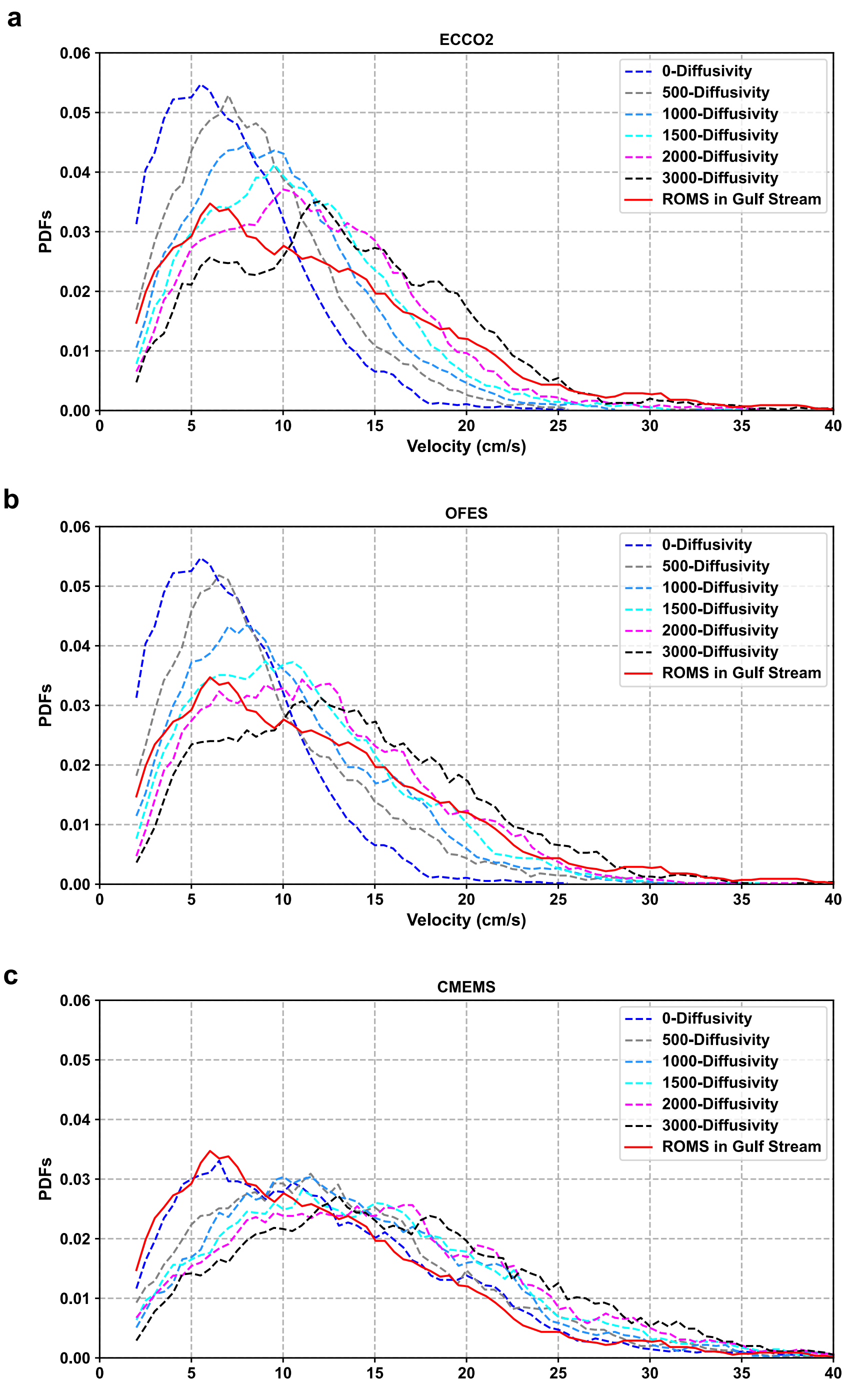

Supplementary Fig. 6 | The probability density function (PDF) of absolute velocity from stochastic float clusters with different diffusivity configurations. (a) for ECCO2 simulation, (b) for OFES simulation, (c) for CMEMS GLORYS12 simulation. All floats are deployed only in the Gulf Stream. The red line represents the simulated floats advected by ROMS velocity fields with no diffusivity. Other colored dashed lines represent the simulated floats with diffusivity at 0-3000 m^2s^{-1}, respectively.

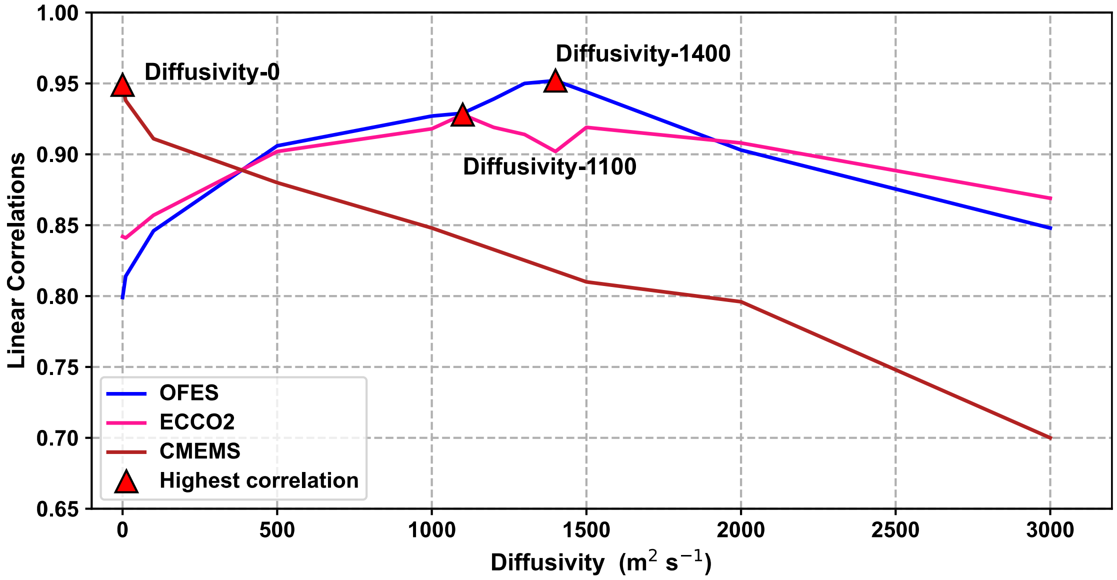

Supplementary Fig. 7 | Correlation of displacements in ROMS with displacements of various diffusivities. The curves in various colors indicate the variation of correlations for different models. The red triangles represent the highest correlations.

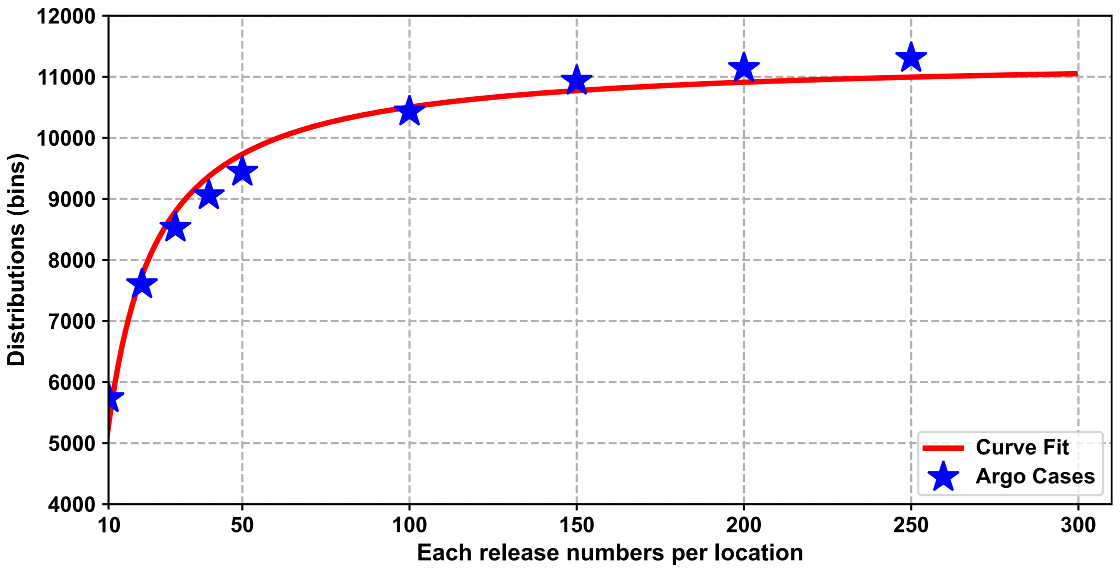

Supplementary Fig. 8 | The convergence test of spatial coverage after simulations completed. The spatial coverage varies with the size of each cluster, indicated by the number of grids on which the simulated floats are located. The simulations of the OFES model in the Gulf Stream are taken as the standard. The size of the grid unit (1/100°×1/100°) is one-tenth of the horizontal resolution of the OFES velocity field. The blue stars represent results from 10 simulation cases, and the red line indicates polynomial fit.

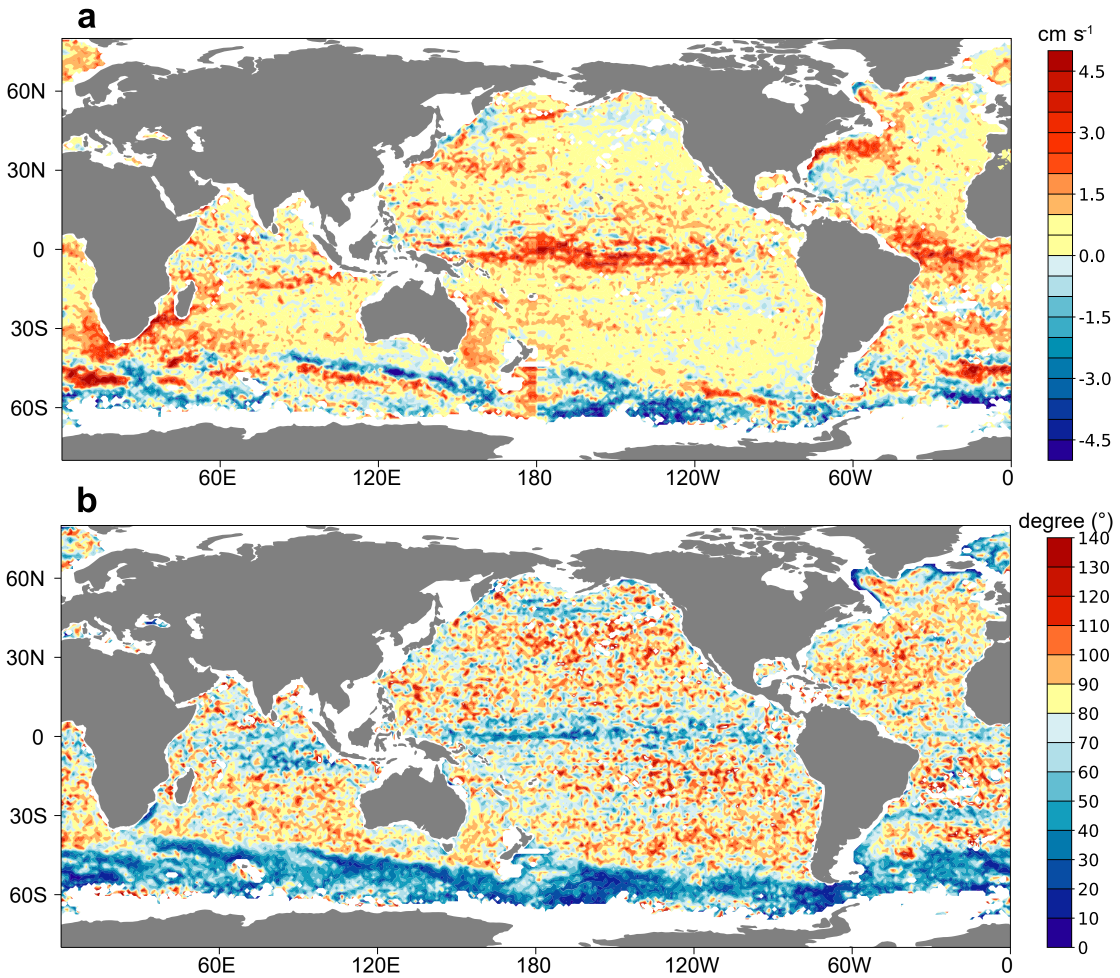

Supplementary Fig. 9 | Global spatial distribution of (a) DOD (Difference of direction) and (b) DOV (Difference of velocity) for OFES in 100-day simulations. The red and blue colors in (a) represent the highest positive and negative DOV, respectively. The red and blue colors in (b) represent the highest and lowest DOD (only positive).

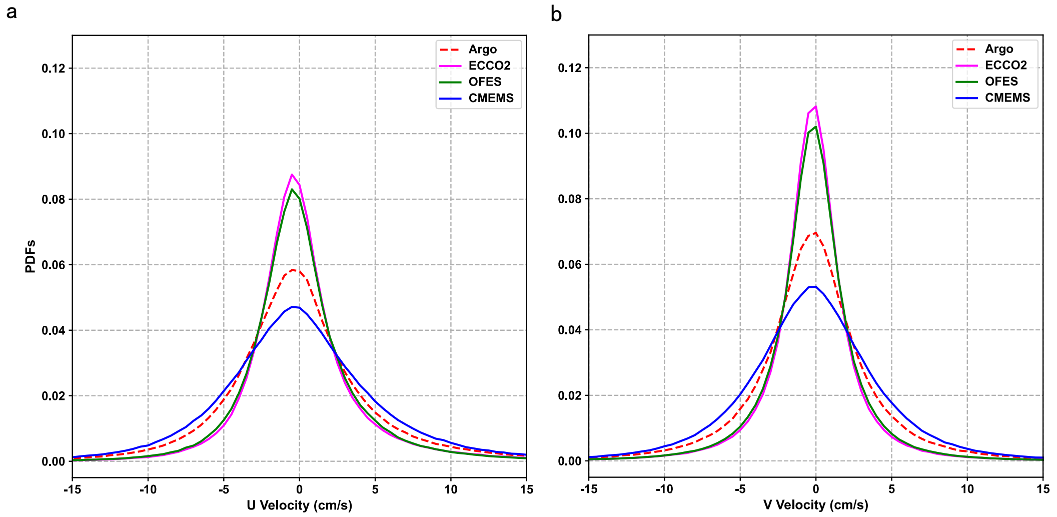

Supplementary Fig. 10 | The probability density function (PDF) of the zonal velocity and meridional velocity from Argo floats and simulated float clusters. (a) Zonal velocity (b) Meridional velocity. The red line represents Argo floats, and the magenta, green, blue line represent simulated floats of ECCO2, OFES, CMEMS GLORYS12, respectively.

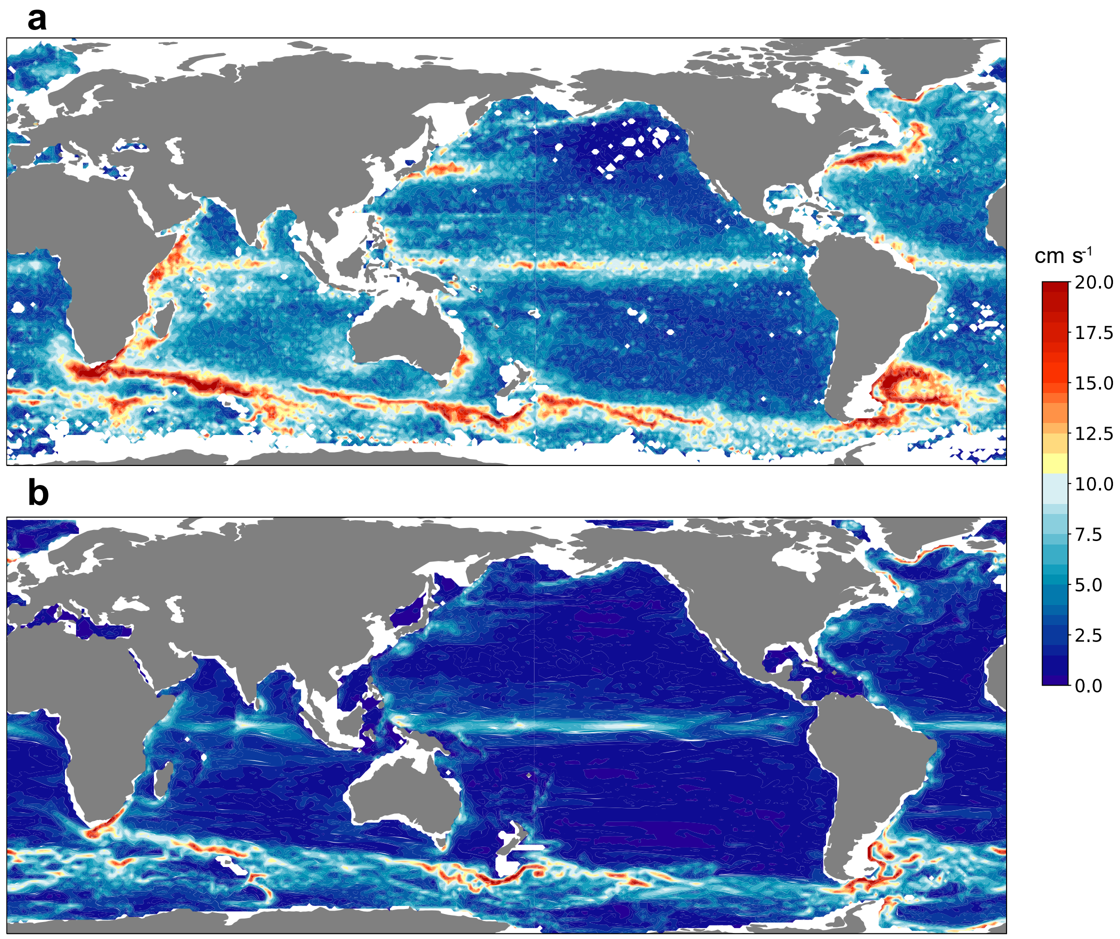

Supplementary Fig. 11 | Global spatial distribution of mean velocity calculated from(a) Argo floats (b) OFES velocity field. The red (blue) color indicates high (low) velocities.

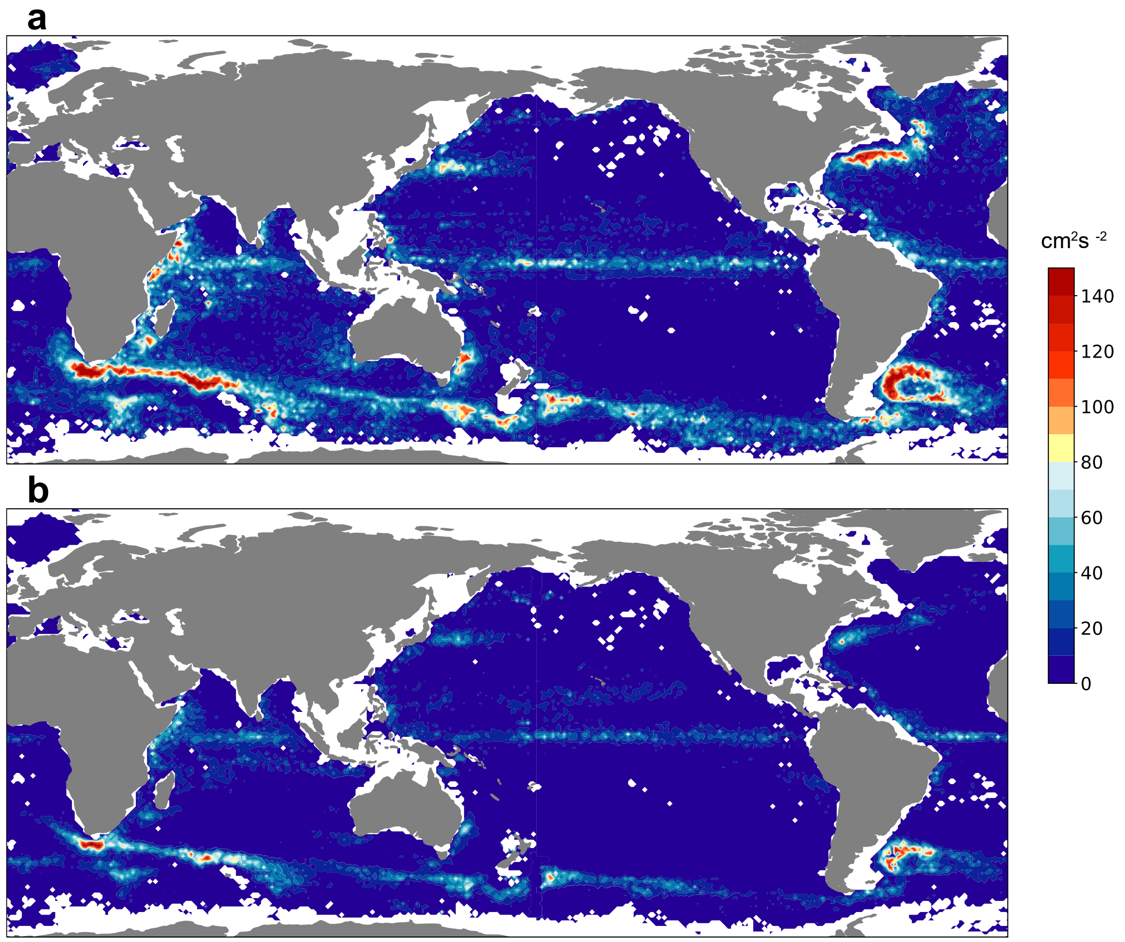

Supplementary Fig. 12 | Global spatial distribution of eddy kinetic energy calculated from (a) Argo floats (b) Simulated OFES floats. The red and blue color indicate high and low eddy kinetic energy distributions, respectively.

Supplementary Table 1 | Percentage of well-understood areas in the global oceans under multiple parameter schemes (calculated by area). The indicators used for analysis here are DOD (Difference of direction), PDOV (Percentage of velocity difference).

Models	DOD (°)	PDOV (%)						
		10%	20%	30%	40%	50%	60%	70%
**ECCO2**	30 °	0.274	0.581	0.885	1.173	**1.415**	1.673	1.869
	40 °	0.575	1.111	1.712	2.281	2.748	3.186	3.503
	50 °	1.154	2.333	3.373	4.497	5.337	6.072	6.592
	60 °	2.160	4.471	6.533	8.559	10.216	11.461	12.327
	70 °	4.324	8.748	12.729	16.373	19.310	21.536	22.977
	80 °	7.944	15.801	22.758	28.954	33.951	37.562	39.915
	90 °	12.016	23.595	34.232	43.539	51.026	56.317	59.850
**OFES**	30 °	0.275	0.553	0.854	1.109	**1.325**	1.538	1.718S
	40 °	0.556	1.194	1.747	2.248	2.647	3.046	3.337
	50 °	1.119	2.303	3.334	4.260	5.052	5.784	6.292
	60 °	2.241	4.489	6.481	8.317	9.786	10.986	11.814
	70 °	4.188	8.457	12.213	15.485	18.171	20.206	21.610
	80 °	7.522	14.916	21.485	27.283	31.880	35.380	37.867
	90 °	11.408	22.434	32.386	41.184	48.159	53.472	57.166
**CMEMS**	30 °	1.288	2.269	2.997	3.482	**3.843**	4.089	4.312
	40 °	2.777	5.106	6.720	8.048	8.969	9.743	10.298
	50 °	4.696	8.766	11.882	14.614	16.657	18.562	19.969
	60 °	6.506	12.475	17.280	21.786	25.463	28.847	31.609
	70 °	7.985	15.378	21.649	27.585	32.626	37.289	41.303
	80 °	9.117	17.526	24.826	31.697	37.745	43.376	48.397
	90 °	9.730	18.661	26.542	33.918	40.536	46.771	52.366

Supplementary Table 2 | Statistics for the indicators of each cluster group. The clustering indicators are DOD (Difference of direction), DOV (Difference of velocity), SD (Separation distance) and SS (Skill score).

	**DOD (°)**	**DOV (cm s^{-1})**	**SD (km)**	**SS (score)**	**Area (%)**
**Cluster 1**	78.549	0.904	44.963	0.375	40.431 %
**Cluster 2**	81.294	-0.790	43.003	0.047	31.074 %
**Cluster 3**	48.825	0.444	49.933	0.535	14.253 %
**Cluster 4**	70.616	2.974	100.686	0.512	14.240 %
**Overall / Average**	69.821	0.883	59.646	0.367	100.000 %
